# Complementary feeding and caregiver sleep: findings from a representative survey in Chongqing, China

**DOI:** 10.3389/fnut.2025.1586206

**Published:** 2025-07-23

**Authors:** Ya Shi, Shengping Li, Junping Chen, Xiangying Su, Zumin Shi, Yan Zhao, Jiaxin Guo, Yong Zhao, Nianrong Wang

**Affiliations:** ^1^School of Public Health, Chongqing Medical University, Chongqing, China; ^2^Research Center for Medicine and Social Development, Chongqing, China; ^3^Department of Children Healthcare, Women and Children’s Hospital of Chongqing Medical University/Chongqing Health Center for Women and Children, Chongqing, China; ^4^Human Nutrition Department, College of Health Sciences, QU Health, Qatar University, Doha, Qatar

**Keywords:** complementary feeding, sleep pattern, caregiver, baby-led weaning, parent-child relationship

## Abstract

**Objective:**

Sleep health and correct complementary feeding are important considerations in public health. This study aims to confirm that correct complementary feeding (CCF) practices are complex and crucial, and they can also influence the development of healthy sleep patterns in infants’ caregivers.

**Methods:**

Using a convenience sampling method, we identified a cohort of Chongqing caregiver-infant pairs (CQ CG-Inf P). Caregivers’ sleep conditions were primarily collected through self-reports, while complementary feeding practices were gathered using the Complementary Food Guide Tool. We employed multiple regression and subgroup analysis to explore the relationship between these factors.

**Results:**

Of the 1,230 respondent pairs, 82.6% of infants were cared for by their mothers, 22% received CCF, and 56.3% of caregivers were housewives. Multivariate logistic regression analyses, both before (Model 1) and after (Model 2) adjustment for socio-demographic characteristics and health conditions of both children and caregivers, consistently indicate that CCF is inversely associated with the establishment of longer sleep patterns among caregivers. The results of the subgroup analysis revealed that the relationship between caregivers’ CCF and long sleep patterns was not influenced by the interaction of caregivers’ basic demographic factors.

**Conclusion:**

This study showed that caregivers providing CCF may face challenges in establishing long sleep patterns. By examining diverse feeding indicators, this research advances understanding in this field. More intuitive training on complementary feeding guidelines can support caregiver sleep health and enhance parent-child interactions.

## Graphical Abstract

Adherence to Complementary Feeding Guidelines Mitigates Abnormal Sleep Patterns in Caregivers. 
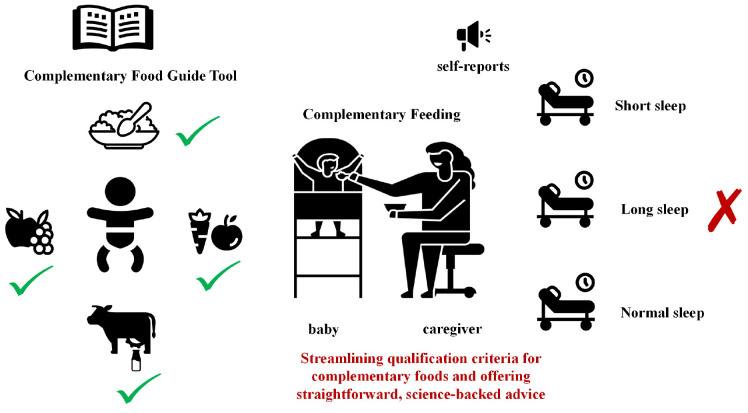

## Introduction

The first 1,000 days of life are crucial for a child’s physical and cognitive development, with the complementary feeding period (6–24 months) playing a key role in promote sleep health (1). The World Health Organization (WHO) recommends introducing complementary foods at six months of age (2). The timing of complementary food introduction and the type of milk feeding have been associated with infant sleep patterns (3). Recent studies have suggested that early introduction of complementary foods (before 4 months of age) or baby-led weaning may help reduce nighttime sleep disruptions (4). However, other studies have reported no significant association between the timing of complementary feeding and infant sleep duration.

Previous studies showed that infant sleep quality is closely intertwined with maternal sleep quality (5, 6). Infant sleep patterns may be influenced by caregiver-led complementary feeding practices, including the timing, content, and frequency of food introduction, suggesting a potential pathway through which feeding practices relate to caregiver sleep. However, few studies have examined how correct complementary feeding (CCF) practices might influence caregiver sleep, prompting our investigation into this underexplored link (7, 8).

Complementary feeding practices, including the timing, frequency, and types of foods introduced, may contribute to sleep disturbances among caregivers during the early postnatal period, especially mothers (9, 10). Feeding practices often contribute to nighttime awakenings and can disrupt caregiver sleep (11). Up to 71% of mothers report poor sleep quality postpartum, with many sleeping less than seven hours per night (12, 13). Low sleep efficiency and frequent night awakenings are associated with increased risks of depression, anxiety, and disrupted mother-infant bonding (14, 15). The mechanisms underlying the relationship between complementary feeding and maternal sleep disturbances may involve multiple pathways. One plausible explanation is Abidin’s parenting stress model (16), which posits that elevated levels of parenting stress, particularly maternal stress, may lead to negative outcomes for children (e.g., emotional unresponsiveness) or the caregiver (e.g., sleep disruption) (17, 18). Additionally, the types of complementary foods and feeding habits (e.g., feeding before sleep) can affect infant sleep, which in turn strongly influences maternal sleep patterns (8).

In light of these findings, this study aims to explore the association between complementary feeding and caregiver sleep, using data from a representative sample in Chongqing, China. We hypothesize that while appropriate complementary feeding practices benefit infant nutrition, they may increase caregiving feeding burdens and adversely affect caregiver sleep.

## Materials and methods

### Study design and participants

The CQ CG-Inf P is an ongoing, prospective, population-based cohort study that recruited 4,093 caregiver-infant pairs (0–36 months) between 2024 and 2025 using a convenience sampling approach. The primary aim is to examine how changes in infant feeding practices are associated with caregiver sleep trajectories over time. The caregiver-infant pairs were included adhering to specific inclusion criteria, (i) Caregivers who voluntarily agreed to participate in this study and signed an informed consent form, (ii) Children aged 0–36 months and their primary caregivers, (iii) Caregivers who did not experience serious complications or comorbidities during pregnancy, (iv) Caregivers who were not affected by mental health conditions that impacted communication. Exclusion criteria encompassed, (i) Children who were officially diagnosed by a medical institution with serious gastrointestinal diseases, such as gastric ulcers or active bleeding. (ii) Mothers or children whose ages did not meet the specified criteria. (iii) Mothers who had mental health conditions, as officially diagnosed by a medical institution, that interfered with the completion of subjective questionnaires. Participants completed a self-reported questionnaire under the guidance of uniformly trained investigators. The questionnaire covered information on caregivers, infant sleep patterns, and feeding practices. The CQ CG-Inf P received ethics approval from the Chongqing Health Center for Women and Children Research Ethics Committee (REC reference no. 2024-083). All participants provided written informed consent for the study.

In the current analysis, we initially included 2,755 caregiver-infant (6–24 months) pairs at baseline. After excluding infants with abnormal birth information (gestational age>42^+6^ weeks, birth weight < 1,000 g, and length < 19 cm; *n* = 245), questionnaires not completed by the primary caregiver (*n* = 519), and missing data on caregiver feeding practices or sleep conditions (*n* = 761), a total of 1,230 pairs were included in the final analysis.

The study benefited from oversight and support from the Chongqing Maternal and Child Health Hospital within the National Maternal and Child Health Monitoring System. Project leaders facilitated expert inspections, supervision, and evaluation of sampling institutions to ensure adherence to rigorous quality control measures. Every sampled district designated a primary project leader responsible for conducting regular quality checks and evaluations of survey activities within their respective communities. Field personnel comprised dedicated project investigators, typically pediatricians specializing in child health, who underwent centralized training to standardize data collection procedures. This methodological framework ensures the reliability and validity of study outcomes through meticulous sampling, robust data collection practices, and stringent quality assurance protocols throughout the research continuum.

### Assessment of sleep patterns in the caregiver

Participants were asked to report the average number of hours that they slept a day on weekdays and weekends, respectively, during the last 1 month. Due to the weekend effect, sleep duration on weekends differs from that on weekdays (19). Sleep duration was assessed by a self-reported questionnaire with the following questions: “What is the average number of hours you have slept on weekdays in the last month?” and “What is the average number of hours you have slept on weekends in the last month?” To achieve the sensitivity analysis, the average sleep time is obtained by the following formula: (weekday sleep time * 5 + weekend sleep time * 2)/7. Sleep duration was classified into three categories: (i) short sleep (SS, < 6 h), (ii) normal sleep (NS, 7–8 h), and (iii) long sleep (LS, ≥ 9 h), based on established criteria from prior studies and aligned with typical sleep patterns observed in the Chinese population (20).

### Assessment of CCF in children aged 6–24 months

Complementary feeding status was evaluated using the Complementary Food Guide Tool (CFGT), developed by the Early Childhood and Early Development Committee of the United Nations Children’s Fund (UNICEF) (21, 22) and the Department of Maternal and Child Health of the National Health Commission (23). Correct complementary feeding (CCF) practices were defined based on the above-mentioned guidelines as the introduction of complementary foods at 6 months of age, along with the provision of at least four food groups per day. These food groups include animal-based products, eggs, dairy, grains, and vegetables or fruits. The CFGT contains questions concerning 7 domains, including 1. Meat/liver/animal blood/fish and shrimp; 2. Eggs; 3. Animal milk and milk products other than breast milk; 4. Staple foods (rice/noodles/potatoes); 5. Legumes/nuts; 6. Dark-colored vegetables/fruits rich in vitamin A (e.g., orange-yellow fruits and veggies, carrots, squash); 7. Other vegetables and fruits. The caregiver completed the questionnaire independently by reporting the frequency of consumption for a total of 7 food items over the past 24 h, with response options including “Not yet,” “One time,” “Two times,” “Three times,” or “Four times.” Then, to enable sensitivity analyses so that caregivers did not misinterpret the frequencies and influence the results, we standardized the frequency of 1–4 times a day as “Yes, having added.” The scores were calculated and added up in each domain, with a “Yes, having added” multiplied by 1 point and a “Not yet” multiplied by 0 points.

To evaluate the validity of the CFGT in this study population, we conducted internal consistency and construct validity tests based on the collected survey data. The CFGT demonstrated good internal consistency, with a Cronbach’s alpha of 0.85. Construct validity was supported by an exploratory factor analysis, with a Kaiser–Meyer–Olkin (KMO) measure of 0.78, indicating satisfactory validity in the context of assessing complementary feeding practices among children aged 6–24 months in Chongqing, China.

### Covariates

This study utilized in-person interviews to complete the questionnaire, collecting socio-demographic data on caregivers and infants as covariates, including caregiver’s age (years), sex (male or female), and baby’s age in month (6–11 months or 12–24 months), baby’s gestational age (24, 25) [preterm newborns (27^+0^ to 36^+6^ weeks) or full-term newborns (37^+0^ to 42^+6^ weeks)], caregiver’s educational level (primary school and below, or junior high school and technical secondary school or college degree and above), family foster model (two parent rearing, single parent rearing, mixed parenting), primary caregiver (baby’s mother or other caregivers) is a relevant factor influencing infant feeding and caregiver sleep patterns (26, 27). To be specific, grandparent-led care is associated with suboptimal feeding and nutritional outcomes, while maternal caregiving, though potentially more responsive, may be affected by psychological stress and job (28). In addition, the covariates studied included mother’s job and baby’s nighttime awakenings status (≤ 3 times or > 3 times) and other infant feeding indicators, e.g., baby-led weaning (BLW), type of milk feeding (mainly breastfed, or mixed feeding, or mainly artificial feeding). Baby-Led Weaning (BLW) was first introduced by Moran and Dykes (29). Most researchers define BLW as an alternative method of infant complementary feeding that encourages self-feeding from around six months of age, rather than traditional parent-led spoon feeding (30). In this study, BLW practice was assessed based on caregivers’ self-reported frequency of spoon-feeding and the texture of complementary foods offered. Following established criteria, infants were categorized into four groups: Primarily Practicing BLW (≤ 10% spoon-feeding), Partially Practicing BLW (> 10% and ≤ 50%), Not Yet Practicing BLW (> 50%), and No Complementary Feeding (not yet introduced to complementary foods) (31).

### Statistical analysis

Stata 18.0 (Stata Corporation, College Station, TX, USA) was used for all statistical analyses. In the descriptive analysis, the continuous variables were described by the mean (standard deviation), and the categorical variables were described by the number (percentage). Categorical covariates were tabulated against different caregivers’ sleep patterns compared by use of the χ^2^ test. Continuous variables were tabulated against different caregivers’ sleep patterns compared by use of the *t*-test. Multinomial Logistic regression analysis was used to investigate associations between being qualified for complementary feeding and caregivers’ sleep patterns. Three models were adopted concerning prior studies on infant feeding and maternal sleep health (32, 33): (i) a crude model without any adjustments; (ii) Model 1, adjusted for key demographic confounders including caregiver’s age, sex, education level, primary caregiver, infant’s age in months, gestational age, and family foster mode; and (iii) Model 2, further adjusted based on Model 1, with the addition of feeding practices and health conditions, including nighttime awakenings, maternal job, and type of milk feeding. In the sensitivity analysis, we selected a one-month range of average sleep duration, focusing on differences between weekends and weekdays to reduce information bias. To explore potential interrelationships among CCF, BLW, and type of milk feeding about caregiver sleep, we used the “corrplot” package in R to visualize their correlation matrix. This helped identify possible collinearity or interaction patterns before multivariable modeling. Results are presented as crude odds ratios (ORs) and adjusted ORs (aORs) with SDs and 95% CIs. *P* < 0.05 was considered to be statistically significant for all statistical analyses.

## Results

### Basic demographic characteristics

The 1,230 caregiver-infant pairs characteristics and categorized into three groups based on the caregiver’s sleep duration: short sleep (*n* = 86), normal sleep (*n* = 688), and long sleep (*n* = 456). are presented in Table 1. The age distribution of the infants was as follows: 49.3% (*n* = 607) were 6–11 months old, and 50.7% (*n* = 623) were 12–24 months old. The gestational age distribution of the infants was as follows: 93.9% (*n* = 1,155) were full-term newborns, and 6.1% (*n* = 75) were preterm newborns. The sex distribution was 51.9% boys (*n* = 638) and 48.1% girls (*n* = 592). The caregivers of infants were 34.10 (SD: 10.4) years old on average, with 98.9% females, and the vast majority of caregivers (86.70%) had been educated at the level of Junior high school diploma or above. Approximately half of the caregivers (67.60%) opted for artificial feeding as the primary method, and the majority (71.20%) had not yet adopted BLW for infant feeding. The vast majority of infants (78.0%) did not meet the CCF criteria, and only 22% received CCF. Additionally, 90.1% of the infants’ nighttime awakenings three times or fewer per day.

**TABLE 1:** Characteristics of the study population by sleep patterns.

Variable^2^	All participants	Short sleep	Normal sleep	Long sleep	*p*-value^1^
	*N* = 1,230	*N* = 86	*N* = 688	*N* = 456	
Infant’s age in months					0.80
6–11 months	607 (49.3%)	45 (52.3%)	335 (48.7%)	227 (49.8%)	
12–24 months	623 (50.7%)	41 (47.7%)	353 (51.3%)	229 (50.2%)	
Infant’s sex					0.56
Boys	638 (51.9%)	40 (46.5%)	357 (51.9%)	241 (52.9%)	
Girls	592 (48.1%)	46 (53.5%)	331 (48.1%)	215 (47.1%)	
Gestational age					0.80
Preterm newborns	75 (6.1%)	4 (4.7%)	44 (6.4%)	27 (5.9%)	
Full-term newborns	1,155 (93.9%)	82 (95.3%)	644 (93.6%)	429 (94.1%)	
Caregiver’s sex					0.048*
Male	13 (1.1%)	0 (0.0%)	4 (0.6%)	9 (2.0%)	
Female	1,217 (98.9%)	86 (100.0%)	684 (99.4%)	447 (98.0%)	
Caregiver’s age	34.1 (10.4)	36.1 (12.5)	34.3 (10.1)	33.6 (10.3)	0.11
Primary caregiver					0.30
Baby’s mother	214 (17.4%)	20 (23.3%)	114 (16.6%)	80 (17.5%)	
Other caregivers^4^	1,016 (82.6%)	66 (76.7%)	574 (83.4%)	376 (82.5%)	
Mother’s job field					< 0.001**
Housewife	693 (56.3%)	37 (43.0%)	364 (52.9%)	292 (64.0%)	
Medical industry	111 (9.0%)	5 (5.8%)	72 (10.5%)	34 (7.5%)	
Other occupations^4^	426 (34.6%)	44 (51.2%)	252 (36.6%)	130 (28.5%)	
Family foster model					
Two-parent rearing	591 (48.2%)	37 (43.0%)	362 (52.6%)	192 (42.1%)	0.006*
Single-parent rearing	65 (5.3%)	4 (4.7%)	30 (4.4%)	31 (6.8%)	
Mixed parenting	570 (46.5%)	45 (52.3%)	296 (43.0%)	233 (51.1%)	
caregiver’s education					< 0.001**
Primary school and below	163 (13.3%)	10 (11.6%)	88 (12.8%)	65 (14.3%)	
Junior high school	374 (30.4%)	35 (40.7%)	174 (25.3%)	165 (36.2%)	
Technical secondary school	314 (25.5%)	16 (18.6%)	183 (26.6%)	115 (25.2%)	
College degree and above	379 (30.8%)	25 (29.1%)	243 (35.3%)	111 (24.3%)	
Type of milk feeding					0.63
Mainly breastfed	283 (23.0%)	19 (22.1%)	156 (22.7%)	108 (23.7%)	
Mixed feeding	116 (9.4%)	12 (14.0%)	65 (9.4%)	39 (8.6%)	
Mainly artificial feeding	831 (67.6%)	55 (64.0%)	467 (67.9%)	309 (67.8%)	
CCF^3^					0.027*
No	959 (78.0%)	73 (84.9%)	518 (75.3%)	368 (80.7%)	
Yes	271 (22.0%)	13 (15.1%)	170 (24.7%)	88 (19.3%)	
BLW^3^					0.18
Not yet practicing BLW	876 (71.2%)	71 (82.6%)	487 (70.8%)	318 (69.7%)	
Partially practicing BLW	117 (9.5%)	2 (2.3%)	67 (9.7%)	48 (10.5%)	
Primarily practicing BLW	176 (14.3%)	8 (9.3%)	99 (14.4%)	69 (15.1%)	
No complementary feeding	61 (5.0%)	5 (5.8%)	35 (5.1%)	21 (4.6%)	
Nighttime awakenings					0.84
≤ 3 times	1,116 (90.7%)	77 (89.5%)	627 (91.1%)	412 (90.4%)	
> 3 times	114 (9.3%)	9 (10.5%)	61 (8.9%)	44 (9.6%)	

^1^*p*-values are based on *t*-tests for continuous variables and chi-squared tests for categorical variables, *P < 0.05, **P < 0.001.

^2^Values are shown as numbers (%), mean ± standard deviation, or median (interquartile range).

^3^CCF, correct complementary feeding; BLW, baby-led weaning.

^4^Other caregivers, referring to individuals other than the baby’s mother who serve as the infant’ s primary caregiver. Other occupations, indicating maternal employment in fields other than being a housewife or working in the medical industry.

### Analysis of the correlations between CCF and the baby’s feeding indicators

**FIGURE 1:**
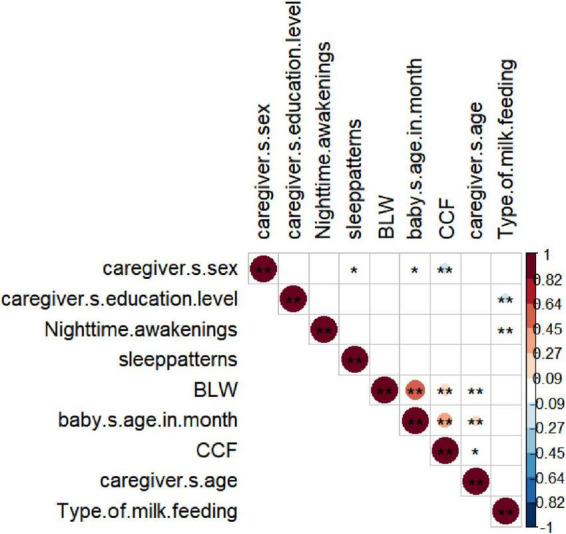
Correlations between CCF and baby’s feeding indicators. Heatmap showing Spearman’s correlations of CCF with caregiver sociodemographic characteristics, infant’s disease status, and feeding indicators. Negative correlations are depicted in blue, and positive correlations are shown in red. The intensity of the colors represents the strength of the correlation, with dark shades indicating strong positive or negative correlations. Symbols ** and * represent the correlation between two factors with *p*-values < 0.01 and < 0.05, respectively.

Positive correlations between CCF and BLW (correlation = 0.20, *p* < 0.01). However, no significant association was found between CCF and the type of milk (see Figure 1).

### Associations between CCF and caregivers’ sleep patterns

Table 2 shows the relationship between CCF and caregivers’ sleep patterns. In the crude model, the association between long sleep patterns and CCF achieved significance when compared to the normal sleep group of the caregivers (OR = 0.73, 95% CI: 0.55, 0.97, *P* < 0.05). This significance persisted in model 1 (OR = 0.69, 95% CI: 0.50, 0.95, *P* < 0.05), model 2 (OR = 0.71, 95% CI: 0.52, 0.98, *P* < 0.05). Additionally, we did not reveal a statistically significant correlation between correct complementary feeding for infants and short sleep patterns in caregivers (*P* > 0.05, crude model, models 1, 2).

**TABLE 2:** The associations between CCF and caregivers’ sleep patterns.

	Crude model^a^		Model 1^b^		Model 2^c^	
CCF	SS vs. NL	LS vs. NL	SS vs. NL	LS vs. NL	SS vs. NL	LS vs. NL
	OR (95% Cl)	OR (95% Cl)	OR (95% Cl)	OR (95% Cl)	OR (95% Cl)	OR (95% Cl)
No	Ref	Ref	Ref	Ref	Ref	Ref
Yes	0.54 (0.29, 1.00)	0.73 (0.55, 0.97)*	0.55 (0.29, 1.05)	0.69 (0.50, 0.95)*	0.53 (0.28, 1.01)	0.71 (0.52, 0.98)*
*P*-value	0.051	0.032	0.070	0.022	0.057	0.036

*P-value < 0.05.

*^a^*Crude model: no covariates were adjusted.

*^b^*Model 1: Caregiver’s age, sex, education level, infant’s age in months and family foster model, gestational age, and primary caregiver were adjusted.

*^c^*Model 2: Additionally add infant’s nighttime awakenings, mother’s jobs, and type of milk feeding were adjusted. CCF, correct complementary feeding; OR, odds ratio; CI, confidence intervals; SS, short sleep, average sleep duration < 6 h. NS, normal sleep, average sleep duration from 7 to 8 h. LS, long sleep, average sleep duration ≥ 9 h.

### Subgroup analyses of the CCF of infants with the caregiver’s long sleep pattern in this survey

Table 3 presents the results of the interaction analyses between CCF and caregivers’ sex, education, infant’s age in months, mother’s job, family foster model, and nighttime awakenings revealed that all *P*-values did not reach the significant level (*P* for interactive > 0.05).

**TABLE 3:** Subgroup analyses of CCF of infant with the caregiver’s long sleep pattern in this survey.

Variable	CCF^a^			*P* for interactive
	No	Yes		
		OR	95% CI	
Infant’s age in months				0.983
6–11 months	1.00	0.82	(0.45–1.49)	
12–24 months	1.00	0.69	(0.47–1.01)	
Caregiver’s education				0.803
Primary school and below	1.00	0.77	(0.33–1.80)	
Junior high school	1.00	0.76	(0.44–1.31)	
Technical secondary school	1.00	1.00	(0.52–1.91)	
College degree and above	1.00	0.59	(0.31–1.11)	
Mother’s job				0.196
Housewife	1.00	0.90	(0.59–1.38)	
Medical industry	1.00	0.22	(0.05–0.96)*	
Other occupations^b^	1.00	0.69	(0.40–1.18)	
Family foster model				0.267
Two-parent rearing	1.00	0.95	(0.60–1.50)	
Single-parent rearing	1.00	0.05	(0.00–1.18)	
Mixed parenting	1.00	0.67	(0.43–1.07)	
Nighttime awakenings				0.813
≤ 3 times	1.00	0.79	(0.57–1.10)	
> 3 times	1.00	0.47	(0.13–1.74)	

*P-value < 0.05.

^a^Caregiver’s age, sex education level, infant’s age in month and family foster model, infant’s nighttime awakenings, mother’s jobs, type of milk feeding, gestational age primary caregiver were adjusted.

^b^Other occupations, indicating maternal employment in fields other than being a housewife or working in the medical industry. CCF, correct complementary feeding; OR, odds ratio; CI, confidence intervals.

## Discussion

Sleep health and correct complementary feeding are two important propositions of this study. Complementary feeding is essential for early infant development (34). While guidelines help caregivers practice correct complementary feeding, they can also create extra challenges. Based on the CQ CG-Inf P study, this survey provided a piece of evidence on the associations of baby’s complementary feeding status with caregiver’s sleep pattern. Our study found that the CCF constraint against long sleep formation. Only 19.3% of caregivers in the long sleep patterns provided their children with CCF. Interestingly, this study also found that caregivers who provided CCF had more difficulty establishing long sleep patterns compared to those who within normal sleep patterns. This relationship remained stable after accounting for the effects of the caregiver’s age, sex education level, infant’s age in months and family foster model, infant’s nighttime awakenings, and mother’s job.

Firstly, we found significant differences in caregivers’ education levels across the sleep duration groups. Specifically, 47.7% of caregivers in the short sleep group had a high school diploma or higher, compared to 61.9% in the normal sleep group and 49.5% in the long sleep group (*p* < 0.05). CCF depends on careful consideration of the infant’s age, chewing ability, and the proper sequence, type, and amount of foods introduced–all of which may be affected by the caregiver’s education level. Caregivers with higher education are more likely to follow strict guidelines for complementary feeding, making infants and their own establishing normal sleep patterns. This finding aligns with the results of Stager et al. (35), who noted that sleep duration is significantly related to education level. Additionally, Tang et al. (36) found Americans that adults with a high school diploma or higher had a lower proportion of short or long sleep. Even among the elderly, those with a college degree or higher had a lower likelihood of short or long sleep patterns compared to those with spouses who had less education (37).

Caregivers with higher education may be likely to follow strict guidelines for complementary feeding, making infants and their own establish normal sleep patterns, which may be influenced by cultural norms. The traditional food cultures and views in different education groups in China influenced the pathway of receiving knowledge of infant feeding. Mothers from a low education level were more likely to receive knowledge of infant feeding from family members, friends, or their own experience, and rarely from health institutions (38). Studies on the Chinese multi-ethnic population have also shown that as the mothers’ education increased, the odds of an unqualified Infant and Child Feeding Index decreased (39). Moreover, a considerable proportion of children’s caregivers live in rural China, who have limited knowledge about exclusive breastfeeding and complementary feeding, especially low-education caregivers absent accurate information sources on infant feeding and child nutrition (40). A US prospective cohort study found that the maternal education level was associated with the practice of the timing of complementary feeding introduction; college graduate mothers were more likely to introduce complementary feeding at ≥ 6 months (41). At the same time, a low level of maternal education was also shown to be associated with inappropriate complementary feeding in the Asian population (42). The irrational complementary feeding resulting from low education may cause children adverse symptoms or disease (1, 43), which may increase demands of maternal “night-time parenting,” and in turn affect the quality and condition of the maternal sleep.

Secondly, 82.6% of the caregivers in this study were the mothers of the children, of whom 56.3% were housewives. The proportion of housewives was significantly greater in the long sleep pattern group than in the normal and short sleep pattern groups. Consistent with other research, it is important to clarify that parental sleep is closely linked to infant sleep (44, 45). In China, mothers, as the main caregivers, play a key role in establishing sleep rhythms and dietary patterns for their infants. Paul’s research indicates that infants sleep to consistent parental sleep patterns (46). As the child grows, their eating patterns align more with the parents (47). Iwata et al. (48) found that disorganized sleep in early infants increases maternal anxiety and mental health issues, which can disrupt solid food introduction and prevent the development of longer sleep patterns, which can be explained by Abidin’s parenting stress model (16). Several studies have shown that irrational complementary feeding may cause children adverse symptoms or disease, including food allergy, bloating, celiac disease, etc (1, 43). These symptoms may increase the demands of maternal “night-time parenting,” and affect the quality and condition of the maternal sleep. Moreover, the professional identity of healthcare workers hinders the establishment of long sleep patterns (49). Healthcare mothers’ busy schedules and complex feeding demands can disrupt their sleep patterns. However, this study did not find an interaction effect between maternal occupation and CCF, likely due to the large disparities in sample sizes across categories, which may have affected the accuracy of the results.

Thirdly, we suspect that the lack of BLW may hinder the development of long sleep patterns. BLW allows infants to take an active role in feeding, reducing caregiver burden and supporting better sleep. Brown and Lee (50) in a descriptive study pointed out that mothers who used BLW ensured that eating is a pleasure for both them and the child, and have lower feeding stress. Another review (51) of maternal-infant feeding practices holds that BLW is a coping strategy to reinforce children’s positive responses, such as reducing feeding frequency at night and increasing the length of infant sleep, and to reach “good mothering” status as measured by societal expectations. And then, this strategy may decrease demands of maternal “night-time parenting,” and in turn ensure the quality and condition of the maternal sleep (1). Complementary feeding practices differ by the child’s primary caregiver, with variations observed between grandparent-led and mother-led care due to differences in feeding attitudes, responsiveness, and contextual stressors (26, 27). Therefore, grandparents should minimize their reliance on traditional or experience-based feeding practices and instead adhere to evidence-based guidelines provided by health institutions. Greater emphasis should be placed on emotional support and psychological interventions for infant caregivers. Furthermore, promoting multi-family and multi-member co-parenting models is also essential to alleviate the rising burden on mothers. However, our study found only a weak positive correlation between BLW and sleep patterns. Due to cultural differences, few Chinese parents in our sample practiced BLW, which may have affected the accuracy of this relationship. Future research should further explore the impact of complementary feeding methods and food variety on maternal and infant sleep.

Previous research has primarily focused on how different types of liquid feeding affect infant sleep, with limited attention to caregiver sleep (7, 52, 53). Additionally, studies on complementary feeding have mostly considered only the timing of introduction (54, 55). In contrast, this study examined various feeding indicators. These included a composite measure of appropriate complementary feeding, covering timing, food diversity, and frequency. We also looked at milk feeding types, and complementary feeding methods. Our findings reveal that CCF can hinder the development of long sleep patterns in primary caregivers. This highlights a key gap in research on how complementary feeding affects sleep. These results suggest that policymakers should consider the practical feeding burdens on caregivers and provide more feasible and supportive feeding guidelines.

This study has several limitations. While we included a broad range of feeding indicators, it is not exhaustive. The absence of quantitative data on complementary food intake, may have attenuated age-related variations. Future research should integrate both diversity and quantity to more accurately capture complementary feeding practices. The concept of composite appropriate complementary feeding proposed in this study may offer new insights into the relationship between feeding and sleep. Additionally, although the study population primarily consists of caregivers, over 82.6% are mothers, reflecting the predominant cultural trend in China where mothers bear the main caregiving responsibilities. This cultural context should be considered when generalizing findings to countries with different cultural norms. Finally, sleep data and complementary food intake were self-reported by caregivers, which could introduce information bias. Future research should include data from multiple complementary feeding assessment methods (digital tools, multiple-day 24-h dietary recall assessments) to more accurately assess this relationship (56, 57).

## Conclusion

This study highlights the relationship between complementary feeding practices and caregiver sleep patterns. Caregivers who provided CCF faced challenges in establishing long sleep patterns. Our study theoretically narrows the scientific gap in understanding the relationship between infant complementary feeding and caregiver sleep. Additionally, it explores the links between diverse feeding indicators (BLW, type of milk, and CCF) and sleep, advancing research in this field. Furthermore, this study provides new insights for visualizing complementary feeding guidelines, enhancing practical training on complementary feeding, and promoting parent-child-friendly interactions. Despite these insights, the study is limited by its focus on caregivers within a specific Chinese cultural context and its reliance on self-reported sleep data. To enhance global applicability, future research should explore diverse cultural settings and incorporate objective sleep measures. Additionally, addressing the practical challenges faced by different types of primary caregivers in adhering to feeding guidelines may help tailor interventions and promote better sleep health outcomes for both caregivers and infants.

## Data Availability

The original contributions presented in this study are included in this article/Supplementary material, further inquiries can be directed to the corresponding authors (see Supplementary Data Sheet 1 for details).
