# Supplementary figures and images for: Complementary feeding and caregiver sleep: findings from a representative survey in Chongqing, China
Source: Front Nutr. 2025 Jul 23;12:1586206. doi: 10.3389/fnut.2025.1586206 (PMC12325014; doi:10.3389/fnut.2025.1586206)

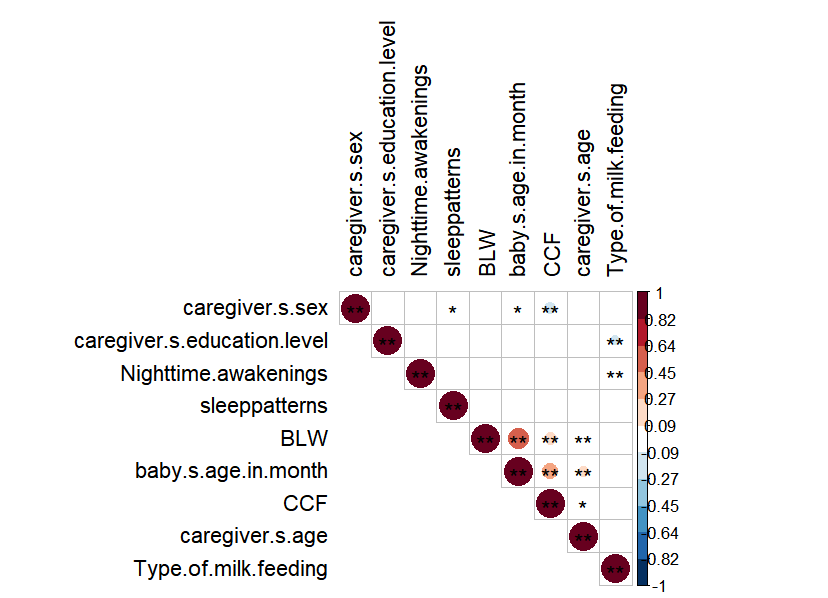

Supplement: Supplementary file 1 [file Data_Sheet_1.zip › Supplementary Material 1(Raw data and Fig.png)/Fig.1 Correlations between CCF and baby’s feeding indicators.png]
